# Central Poststroke Pain: Its Profile among Stroke Survivors in Kano, Nigeria

**DOI:** 10.1155/2017/9318597

**Published:** 2017-09-19

**Authors:** Abdulbaki Halliru Bashir, Auwal Abdullahi, Muhammad Aliyu Abba, Naziru Bashir Mukhtar

**Affiliations:** Department of Physiotherapy, Bayero University Kano, Kano, Nigeria

## Abstract

**Background:**

Central poststroke pain (CPSP) caused by sensory dysfunction of central origin is a disabling condition that significantly affects the quality of life of stroke patients.

**Aim:**

The aim of this study is to determine the clinical profiles and pattern of CPSP among stroke patients in Kano, Nigeria.

**Methods:**

The study was a cross-sectional design involving stroke survivors who were ≥18 years old and with no significant cognitive impairment approved by the Research Ethics Committee of Aminu Kano Teaching Hospital. Participants were assessed using diagnostic criteria form, the douleur neuropathique 4 questions (DN4 questionnaire), and Leeds assessment of neuropathic symptoms and signs (LANNS).

**Results:**

A total of 120 stroke patients participated in the study, in which 6 (5%) were diagnosed with CPSP occurring within the first 3 months in 50% of the participants. The pain characteristics were mainly moderate (83.3%), burning (62.5%), and continuously experienced (66.7%). The frequently affected parts were extremities or occurring as hemisyndrome.

**Conclusion:**

Prevalence of CPSP following stroke is low. The clinical features are variable and can occur at a varied time and different intensities and locations. However, it majorly occurs within the first few months post stroke.

## 1. Introduction

Stroke is now an important public health concern. It is classically characterized as a neurological deficit attributed to an acute focal injury of the central nervous system (CNS) by a vascular cause, including cerebral infarction, intracerebral hemorrhage (ICH), and subarachnoid hemorrhage (SAH) [1]. When stroke occurs, there may be impairment in the brain's control of motor, sensory, and cognitive functions in combination with one another or all. One of the impairments in sensory functions following stroke is central poststroke pain (CPSP).

Central poststroke pain (CPSP) is a constant or intermittent pain reported by patients after stroke that is usually associated with sensory abnormalities such as decreased perception of harmful and sharp stimuli [2]. The symptoms may affect the patients' ability to carry out their activities of daily living, cause emotional disturbances, and decrease the patients' quality of life [3–5].

Although, there remain some mysteries as to the pathophysiology of CPSP, it is believed to be caused by stroke in the region of the thalamus [6] and extrathalamic areas [7]. The thalamus is a relay station for sensory information from all over the body. Subsequently, it was coined initially as thalamic pain syndrome (TPS) by Dejerine and Roussy in 1906 [8]; and so far, there have been different treatments for it. The treatments of CPSP involve pharmacologic or nonpharmacologic treatment. The pharmacologic treatment includes the use of antidepressants [3] and opioids such as morphine [9]. The nonpharmacological treatment includes the use of repetitive transmagnetic stimulation [10], deep brain stimulation [11], and vestibular caloric stimulation [12]. Despite these available treatments, CPSP is a difficult condition as it is usually underreported probably due to payment of attention majorly on motor impairment and spasticity by the clinicians [13]. Furthermore, as stroke is now increasingly becoming a leading cause of disability in the developing countries such as Nigeria [14], there may be likelihood of having increased prevalence of CPSP among the survivors. Therefore, understanding the profiles of any of its symptoms is important for healthcare planning, management, and rehabilitation. The aim of this study is to look at the profiles of CPSP and gender difference in the profiles among stroke survivors in Kano, Nigeria. Thus, the study will answer the research question what is the profile of PSCP among stroke survivors in Kano state, Nigeria. The answer to this question will help clinicians and researcher alike to better understand the profile and extent PSCP in the given population and work for better therapeutic intervention for it.

## 2. Material and Methods

### 2.1. Participants and Recruitments

The study was a cross-sectional (observational) study approved by the Research Ethics Committee of Kano State Hospitals Management Board which caters for all the hospitals under the jurisdiction of the state government. The population of the study was patients with stroke attending outpatient physiotherapy departments at Murtala Muhammad Specialists Hospital (MMSH) and Muhammad Abdullahi Wase Specialist Hospital (MAWSH) in Kano. Participants were sampled using convenience sampling technique using the following inclusion criteria: adult stroke patients who are 18 years of age or above and patients with no significant cognitive impairment (Mini-Mental State Examination (MMSE) >17).

### 2.2. Procedure

The nature and procedure of the study were explained to the participants, and those who signed the informed consent form were interviewed. Information about demographic characteristics of the study participants, stroke condition, and pain were obtained and documented in diagnostic criteria form. The diagnostic criteria form used by Klit and his colleagues were used to assess for participants who are suspected to have CPSP [15]. The criteria are as follows: having other pain other than the ones listed in the form; presence of abnormal sensation, whether the side of the abnormal sensation corresponds with the area of other pain; anatomically probable distribution of the indicated areas of altered sensation or pain that is either unilateral or crossed head/body distribution; and absence of any other obvious cause of the pain such as nociceptive or psychogenic.

Other instruments used in the study include pain assessment record form for collecting the details on the pain characteristics such as onset, description, abnormal sensation and its description, brush for testing dynamic allodynia, toothpick used for testing pinprick hyperalgesia, test tube for heat (40°) and cold (15°) sensation, and alcohol for sterilization. Pinprick and thermal (heat and cold) sensations were tested at the patients' pain location at the interval of 5 minutes each. Additionally, the douleur neuropathique 4 questions (DN4 questionnaire) and Leeds assessment of neuropathic symptoms and signs (LANNS) were also used.

The DN4 questionnaire is a 4-item questionnaire developed by Bouhassira and colleagues [16]. It is a reliable scale that differentiates between neuropathic pain and nonneuropathic pain [17]. Similarly, the LANNS is a valid instrument that distinguishes nociceptive pain from neuropathic pain [18, 19]. It consists of 2 sections: pain questionnaire which has 5 items and sensory testing which has 2 items [18].

### 2.3. Data Analysis Procedure

The demographic characteristics of the study participants and their CPSP clinical profiles were analyzed using descriptive statistics of mean, frequency, and percentage. The gender difference in the clinical profiles was analyzed using Mann–Whitney *U* test. All analyses were carried out using SPSS version 16.

## 3. Result

A total of 124 patients were contacted for the study; out of this, 4 refused to participate. One hundred and twenty patients were interviewed in which 20 were suspected to be having CPSP. After sensory and clinical examinations, 6 patients were diagnosed with CPSP corresponding to an incidence of CPSP of 5% in the study population.

Out of the 120 stroke patients assessed, 57 (47.5%) were male and 63 (52.5%) were female with mean age of 55.8 ± 10.9. For the participants diagnosed with CPSP, 4 (66.7%) were male and 2 (33.3%) were female with mean age of 59.3 ± 9.4 (47–73). All of the participants with CPSP had ischemic stroke, and one had recurrence of the stroke within 1 year. See Table 1 for the demographic characteristics of the participants diagnosed with CPSP.

The average period of the onset of stroke to the time of the study was 11 ± 5.7 months, and average period of pain onset from stroke onset was 4.2 ± 4.3 months. One (16.7%) of the participants started experiencing chronic pain immediately after stroke; in 3 of the participants (50%), the pain onset was within 3 months after onset of stroke; and in 2 of the participants (33.3%), the pain was after 6 months. Majority of the participants were experiencing moderate pain (83.3%) and the rest, severe. Four (66.7%) of the participants reported the pain to be continuous and had experienced change in pain from its onset. Burning sensation (62.5%) is the most reported pain descriptor followed by electric shock. At least 2 (33.3%) of the participants had more than one pain descriptor, and 1 (16.7%) had 3 pain descriptors. All the participants had 1 or 2 other associated poststoke pains, 50% had painful shoulder, and 20% had spasticity related pain. See Table 2 for more detail.

The main aggravating factors reported were psychological stress, contact to cold/heat, and movement of the limb. The average DN4 questionnaire and LANNS scores were 5.5 ± 1.0 and 16.2 ± 2.1, respectively, indicating that neuropathic mechanisms are contributing or responsible to the patient symptoms. Pain location and distribution were multifocal (90.9%) in almost all the participants. The main sensory abnormalities were tactile allodynia (35.7%) and pinprick hyperalgesia (35.7%). About 33% of the participants had 3 sensory abnormalities, and all had more than one sensory abnormality as shown in Table 3. About 50% of the participants were reported after sensation during the examination. Pins and needles were the common abnormal sensation descriptor reported by 4 (44.4%) participants and tingling in 3 (33.3%) participants. At least 2 of the participants had 3 sensation descriptors. See Table 4 for more details.

For the difference in the clinical characteristics between male and female participants diagnosed with CPSPS, Mann–Whitney *U* test revealed that there was no significant difference (*p* > 0.05). See Table 3 for more details of the analysis.

## 4. Discussion

The aim of this study was to find out the profiles of CPSP in Kano, Nigeria. The result of the study showed a CPSP prevalence of 5% which is within the range of 1–12% reported in the literature by Klit and colleagues and Kong and colleagues [15, 20–22]. Whereas, the study by Klit and colleagues was a population based with a very large sample, and the present study was hospital based. Consequently, it may be difficult to categorically state that the prevalence rate well represents that of Kano population especially that it is in a developing country where there are still many challenges with stroke rehabilitation as many stroke survivors may not be reported or get access to quality care [23–25]. However, the low prevalence of CPSP reported in the present and previous studies should not take precedence over the burden it may impose on the patients in terms of interfering daily activities and has a significant impact on their health-related quality of life [20]. Therefore, very effective therapeutic interventions are needed to help these patients.

The result also showed that the duration of CPSP onset varies greatly. In the present study, about 50% of the participants had pain onset within the first three months and 16.7% immediately after stroke. In consistent with our findings, previously, it was reported that CPSP occurs between 3 and 6 months post stroke [26]. However, it was said to be commonest within the first few months post stroke [27]. Consequently, it may be very advisable if clinicians are watchful for the signs and symptoms of CPSP at any time post stroke. This could help to forestall its attending effects on the well-being of the patients.

Occurrence of CPSP may depend on gender. In this study, CPSP is more prevalent in men than in women. However, it has been argued that gender is not a reliable predictor of CPSP as it may be more prevalent in either men or women [7]. Additionally, the pain intensity in the study was moderate in about 80% of participants which is similar to the findings of de Oliveira and colleagues [28]. Similarly, the location and distribution were multifocal in most of the participants. However, pain intensity, location, and distribution can change. For instance, pain intensity can change from moderate to severe especially that the onset can also vary [29, 30]. Thus, it is important for the clinicians to attend to the pain at the onset to help prevent it from changing to severe form. This is because, in this study, about 80% of the participants reported change in the pain since its onset in either intensity or distribution or both. Similar pain characteristics were also reported by Klit and colleagues [15].

Central poststroke pain (CPSP) can occur alongside other pain symptoms, though it is difficult to distinguish between CPSP with other pain syndromes [31]. In this study, each of participants had 1 or 2 other poststroke pains occurring concomitantly. Out of these, shoulder pain accounted for about 50% of the pains. Previous studies had similarly showed the prevalence of shoulder pain in CPSP patients to be 30–40% [30, 32]. Thus, pain management should be an integral part of stroke rehabilitation. Additionally, we found allodynia to be the most abnormal sensation accounting for 62.5% which is consistent with the findings of a previous study [15]. However, the type of allodynia CPSP patients may have depend on the lesion location in the thalamus [33]; and those with normal tactile detection thresholds may experience tactile allodynia significantly more often than those with tactile hypoesthesia [34]. Furthermore, all of our study participants had high DN4 and LANNS scores. Similarly, it was previously reported that most of the patients with CPSP had high DN4 questionnaire scores [15]. The DN4 is very sensitive at selecting a subgroup of CPSP patients with more severe pain and sensory abnormalities [35]. This suggests that the participants' symptoms are results of neuropathic mechanism.

Poststroke central pain can have psychological impact on the patients, reduces sleep quality, and interferes with activities of daily living. Previously, pain in stroke patients was reported to result in sleep disturbance and general well-being [36]; and pain, especially when it is chronic, can be associated with depression [37]. These can eventually affect health-related quality of life [20]. Additionally, the risk factors for CPSP include ischemic stroke (similarly, all the participants in this study had ischemic stroke, though the diagnosis was done only using clinical presentations as radiological reports of the participants were not available), impaired limb motor function, and presence of sensory abnormalities [15, 37]. Thus, prevention of risk factors of ischemic stroke, pain management and rehabilitation of sensory, and motor function may help prevent the incidence of CPSP or reduce its psychosocial and physical effects of CPSP on the patients. However, the present study is limited by its sample size.

## 5. Conclusion

Prevalence of CPSP following stroke is low. The clinical features are variable and can occur at varied times and different intensities and locations. However, it majorly occurs within the first few months post stroke. Therefore, clinicians are advised to be on the watch out for its signs and symptoms in order to help forestall any possible burden it will impose on the patients.

## Figures and Tables

**Table 1:** Summary of the demographic characteristics and pain scores of participants diagnosed with CPSP.

Variable	*N*	%
Patients diagnosed with CPSP	6 out of 120	5
Male with CPSP	4 out of 57	7
Female with CPSP	2 out of 63	3.2
Mean age	59.3 ± 9.4	
Mean period of onset of stroke	11 ± 5.7 months	
Mean period of pain onset	4.2 ± 4.3 months	
Mean VAS score	6.2 ± 1.2	
Average DN4 scores	5.5 ± 1.0	
Average LANNS scores	16.2 ± 2.1	

*N*: frequency; %: percentage; DN4: douleur neuropathique 4 questions; LANSS: Leeds assessment of neuropathic symptoms and signs.

**Table 2:** Showing pain characteristics of participants with CPSP.

	*N*	%
*Pain locations*		
Hemibody	1	9.1
Inferior upper limb	2	18.2
Inferior lower limbs (leg & foot)	2	18.2
Superior upper limb (arm)	1	9.1
Lower limbs	2	18.2
Hands	1	9.1
Shoulder	1	9.1
*Pain onset*		
Immediately after stroke	1	16.7
Within 3 months after stroke	3	50
Six months after stroke	2	33.3
*Pain intensity (VAS scores)*		
Moderate (4–7)	5	83.3
Severe (8–10)	1	16.7
*Pain frequency*		
Continuous	4	66.7
Intermittent	2	33.3
*Pain change from onset*		
Yes	4	66.7
No	2	33.3
*Other poststroke pains*		
Shoulder pain	5	50
Headache	1	10
Spasticity-related pain	2	20
Joint pain	2	20

*N*: frequency; %: percentage.

**Table 3:** Summary of Mann–Whitney *U* test for difference in CPSP pattern across genders.

Variables	Gender	*N*	Mean rank	Sum of ranks	*U*	*p* value
Prevalence	m	4	3.50	14.00	4.00	1.000
Pain onset	m	4	4.38	17.50	0.500	0.100
	f	2	1.75	3.50		
Pain	m	4	3.25	13.00	3.000	0.576
Frequency	f	2	4.00	8.00		
Pain	m	4	2.50	10.00	0.000	0.057
Intensity	f	2	3.50	11.00		
Pain	m	4	3.00	12.00	2.000	0.157
Descriptors	f	2	4.50	9.50		
Sensory	m	4	3.50	14.00	4.000	1.000
Abnormalities	f	2	3.50	7.000		
Sensation	m	4	3.50	14.00	2.000	1.000
Descriptors	f	2	3.50	7.00		
DN4 scores	m	4	3.12	12.50	2.500	0.475
	f	2	4.25	8.50		
LANSS	m	4	4.12	16.50	1.500	0.240
Scores	f	2	2.25	4.50		
Other PSPs	m	4	4.00	16.00	2.000	0.273
	f	2	2.50	5.00		
Pain change	m	4	4.00	16.00	2.000	0.264
	f	2	2.50	5.00		

*N*: frequency; %: percentage; DN4: douleur neuropathique 4 questions; LANSS: Leeds assessment of neuropathic symptoms and signs; PSPs: poststroke pains.

**Table 4:** Showing abnormal pain sensation of the participants.

Variable	*N*	%
*Pain description*		
Burning	5	62.5
Electric shock	2	25.0
Knifelike	1	12.5
*Sensation description*		
Pins and needles	4	44.4
Numb	2	22.2
Tingling	3	33.3
*Sensory abnormalities*		
Allodynia	5	62.5
Pinprick hyperalgesia	5	25.0
Cold hyperalgesia	2	12.5
Heat hyperalgesia	2	12.5

*N*: frequency; %: percentage.
